# Patients with Obstructive Sleep Apnea Display Increased Carotid Intima Media: A Meta-Analysis

**DOI:** 10.1155/2013/839582

**Published:** 2013-08-27

**Authors:** Rashid Nadeem, Michael Harvey, Mukesh Singh, Ahmed Abdullah Khan, Mustafa Albustani, Aaron Baessler, Essam M. Madbouly, Hassan Sajid, Mahnoor Khan, Nayab Navid

**Affiliations:** ^1^Chicago Medical School, Rosalind Franklin University of Medicine and Science, North Chicago, IL 60064, USA; ^2^Pulmonary and Sleep Medicine, James A. Lovell Federal Health Care Center, 3001 Green Bay Road, North Chicago, IL 60064, USA; ^3^Sindh Medical College, Rafiqi H. J. Road, Karachi 75510, Pakistan; ^4^Al-Kindy College of Medicine, University of Baghdad, P.O. Box 47188, Baghdad, Iraq; ^5^McMaster University, 1280 Main Street West, Hamilton, ON, Canada L8S 4L8; ^6^Benedictine University, 5700 College Road, Lisle, IL 60532, USA

## Abstract

*Background*. Obstructive sleep apnea (OSA) is associated with coronary artery disease. Intermittent hypoxia associated with OSA increases sympathetic activity and may cause systemic inflammation, which may contribute to atherosclerosis leading to an increase in the size of carotid intima media thickness (CIMT). *Methods*. PubMed and Cochrane library were reviewed by utilizing different combinations of key words: sleep apnea, carotid disease, intima media thickness, and carotid atherosclerosis. Inclusion criteria were English articles; studies with adult population with OSA and without OSA; CIMT recorded by ultrasound in mean and standard deviation or median with 95% confidence interval; and OSA defined as apnea hypopnea index of ≥5/h. A total of 95 studies were reviewed for inclusion, with 16 studies being pooled for analysis. *Results*. Ninety-five studies were reviewed, while 16 studies were pooled for analysis; since some studies have more than one data set, there were 25 data sets with 1415 patients being pooled for meta-analysis. All studies used ultrasound to measure CIMT. CIMT standardized difference in means ranged from −0.883 to 8.01. The pooled standardized difference in means was 1.40 (lower limit 0.996 to upper limit 1.803, (*P* < 0.0001). *Conclusion*. Patients with OSA appear to have increased CIMT suggestive of an atherosclerotic process.

## 1. Introduction

Obstructive sleep apnea (OSA), a common disorder, is often asymptomatic, and the prevalence of patients with OSA, who do not present clinical syndrome, might be as high as 20–30% in the middle-aged population [1]. OSA is a significant source of morbidity and mortality [2]. OSA is characterized by recurrent episodes of upper airway collapses during sleep. These recurrent episodes of upper airway collapse usually are accompanied by oxyhemoglobin desaturation and terminated by brief arousals which result in marked sleep fragmentation and chronic excessive daytime sleepiness (EDS) [1, 3]. As a result, there are an increased expression of systemic inflammatory markers, a sustained activation of the sympathetic nervous system [4], and derangement in endothelial function [5]. Many of these physiologic and biochemical abnormalities are implicated in the pathogenesis of cardiovascular and cerebrovascular diseases.

Carotid intima media thickness (CIMT) is a surrogate marker for atherosclerotic disease and a means to detect subclinical atherosclerosis [6]. CIMT as measured by B-mode ultrasound represents the combined thickness of the intimal and medial layers of the carotid artery. Despite its limitations, measurement of CIMT is a noninvasive technique and has several advantages over other surrogate markers of atherosclerotic disease.

CIMT can be recorded multiple times without inflicting any physical harm [7]. Patients can also have repeated CIMT measurements done without being exposed to radiation, which is commonly associated with other arterial visualization methods [8]. Lastly, measurement of CIMT uses equipment that is readily available, relatively inexpensive, and not hindered by patient anatomy [9].

Multiple studies have shown that increased CIMT is a valid predictor of the probability of future cardiovascular events and can predict the presence of coronary artery disease (CAD) [10, 11]. OSA is thought to be associated in the pathogenesis of CAD along with other cerebrovascular events. OSA patients are at increased risk of cardiovascular morbidity and mortality, mostly involving systemic hypertension, coronary heart disease, stroke, and heart failure [12]. Oxidative stress, low grade inflammation, and sympathetic activation have been proposed as key mechanisms explaining the increased cardiovascular risk in OSA. Repetitive desaturation-reoxygenation sequences have been shown to generate reactive oxygen species (ROS), impair serum antioxidant capacity, and enhance lipid peroxidation [13]. This oxidative stress is known to damage the vascular endothelium, to promote atherosclerosis, and ultimately to favor the later occurrence of adverse cardiovascular events [13]. Therefore, a direct correlation between OSA and elevated CIMT may be elucidated [14]. However, studies evaluating this relationship have been small and have yielded conflicting results. Therefore, a meta-analysis was performed to assess if there is any direct correlation between OSA and CIMT. 

## 2. Methods 

We performed this review in accordance with PRISMA guidelines for performing meta-analysis. A protocol was prospectively developed, detailing the objectives, criteria for study selection, and approach to assessing the study quality, primary outcome, and methodology. 

### 2.1. Data Source and Study Selection

Studies for review were found searching the PubMed, Cochrane, and EMBASE databases from January 01, 1960 to December 31, 2012. Unpublished data from scientific meetings were not searched since most abstracts do not provide detail data needed for meta-analysis. The searches were conducted using the following keywords: sleep apnea, obstructive sleep apnea, carotid disease, intima media thickness, and carotid atherosclerosis. Abbreviated forms of keywords were also searched to ensure that any relevant sources were not excluded. Multiple authors individually searched for and scored manuscripts for inclusion. Manuscripts were scored in duplicates, and if manuscripts were scored differently by two authors, they were reviewed by a third author to finalize inclusion. 

### 2.2. Studies and Endpoint Definitions

CIMT was selected as a marker for atherosclerotic process associated with OSA based on review of literature. Inclusion criteria defined for study selection were as follows: (1) the study had to be in English; (2) full text manuscripts had to be available; (3) the study had to contain values corresponding to CIMT recorded by ultrasound; (4) the study had to include at least two separate groups with one being subjects diagnosed with obstructive sleep apnea and the other group consisting of subjects without obstructive sleep apnea; (5) OSA was strictly defined as AHI ≥5/h; (6) the study must have reported values in mean and standard deviation or median with 95% confidence interval; (7) subject number for all groups had to be reported; (8) the study must have been performed on adult humans. Studies that did not meet the above criteria were excluded.

### 2.3. Data Extraction and Statistical Analysis

Data was extracted at a study level by a single author and then reviewed by a second author to ensure that no errors were made. CIMT measurements were extracted from studies as mean with standard deviation. Studies with data reported as median with range, mean and standard deviation were calculated utilizing methods outlined by Hozo et al. [15].

Target variable (carotid intima media thickness) was recorded as well as reported demographics (age, gender, and BMI) and confounding factors (AHI, LDL cholesterol, HDL cholesterol, and blood pressure) to evaluate the effect of these parameters on the target by employing subgroup analysis or metaregression.

For studies in which OSA groups were divided based on severity, each set of data was included into the meta-analysis as separate studies. For example, Drager and colleagues [16] divided the OSA subjects into mild, moderate, and severe groups. Therefore, three different sets of data were collected from the study comparing each OSA group to the control. Also, studies in which the additive effects of OSA and another disease process, that is, HTN, metabolic syndrome (MetS) on CIMT, were evaluated; both sets of data were included separately into the meta-analysis. For example, Drager and colleagues [17, 18] measured CIMT on patients diagnosed with hypertension and without hypertension with and without OSA. Similarly, Monneret and colleagues [19] measured CIMT on patients diagnosed with metabolic syndrome, with and without OSA.

Risk for bias was assessed at study level, and, at outcome level and according to our inclusion criteria, multiple studies were excluded from the meta-analysis based on OSA diagnosis designation, as these studies used different cutoff values for AHI to define OSA patients, and some studies did not directly classify their subjects as suffering from OSA. For example, Wattanakit and colleagues [20] as well as Sackett and colleagues [21] divided their subjects into groups with or without sleep disordered breathing, with no reference to OSA. Study selection, data extraction, and statistical analyses were all done in accordance with previously published methodology for meta-analyses. Measurement units of carotid intima media thickness used in the meta-analysis were micrometers (*μ*m). If any of the measurements were not reported in the same standard measurement unit that we used, the value was converted to the micrometers.

### 2.4. Statistical Analysis

The statistical analysis was performed by the Comprehensive Meta-Analysis software package (version CM 2.2, Biostat, Englewood, NJ, USA). Heterogeneity analysis by the Cochran's *Q* statistics for individual end points across all studies was performed. An *I* ^2^ of 25–49% was considered to represent a low level of heterogeneity, 50–74% a moderate level and 75–100% a high level. A two-sided alpha error of less than 0.05 was considered to be statistically significant.

## 3. Results

### 3.1. Literature Search

The literature was ranked according to the Sackett et al's [22] hierarchy of evidence. A total of 95 studies were reviewed for inclusion, and 79 manuscripts were excluded (reviews = 24, case report = 4, expert opinion = 2, non-human studies = 4, non-English paper = 5, non-adult population = 4, study reported no data as two separate groups (OSA and control) = 10, CIMT measured by technology other than ultrasound = 4, study used OSA definition other than AHI >5/hour = 4 studies, diagnosis of sleep apnea was not made by sleep study = 4, only graphical display of data available = 2, and only abstract available with no usable data = 12). Sixteen studies were eventually pooled for analysis. Quality of evidence was low (3B-individual case-control study) for the 16 included studies. 

### 3.2. Overview of Study and Patient Characteristics

Studies provided 25 data sets with a total of 1415 patients pooled for meta-analysis. Key studies of CIMT measurement in patients with and without OSA are outlined in Table 1. 

### 3.3. OSA and CIMT

**Figure 1:**
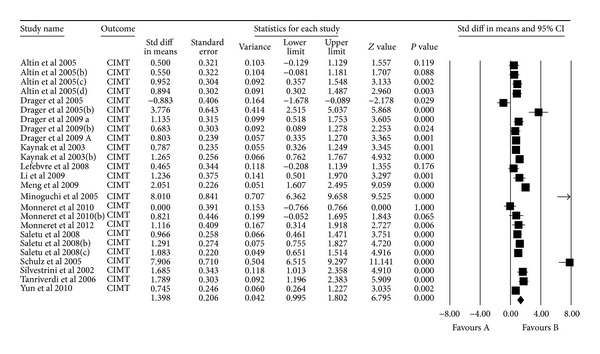
Meta-analysis.

CIMT standardized difference in means ranged from −0.883 to 8.01. The pooled standardized difference in means was 1.40 (LL 0.996 to UL 1.803, *P* < 0.0001) (Figure 1). 

### 3.4. Metaregression to Evaluate the Effect of Confounding Factors

**Figure 2:**
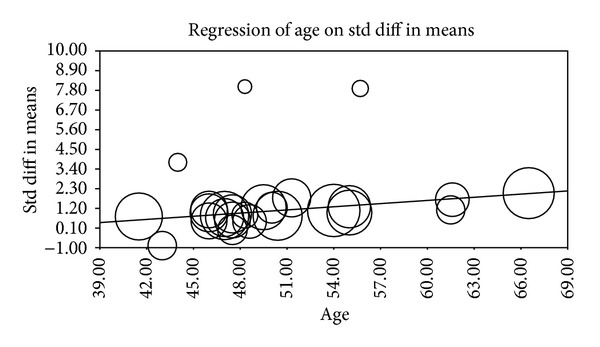
Age metaregression.

**Figure 3:**
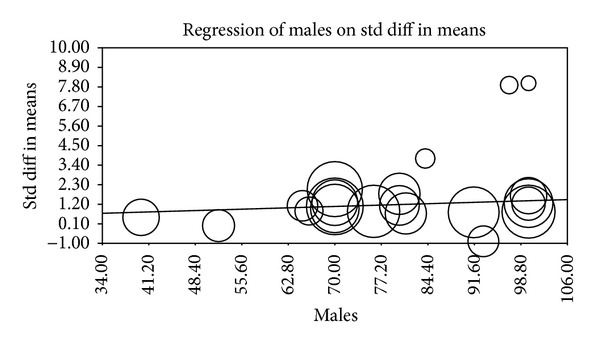
Gender metaregression.

**Figure 4:**
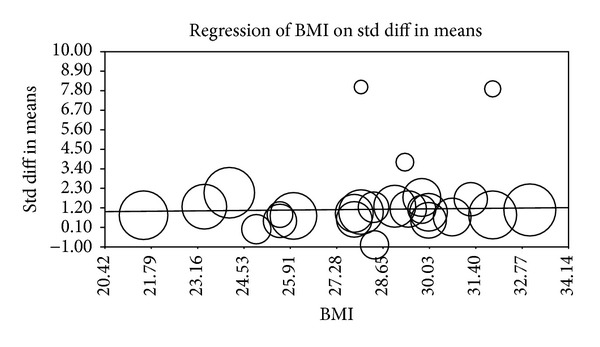
BMI metaregression.

**Figure 5:**
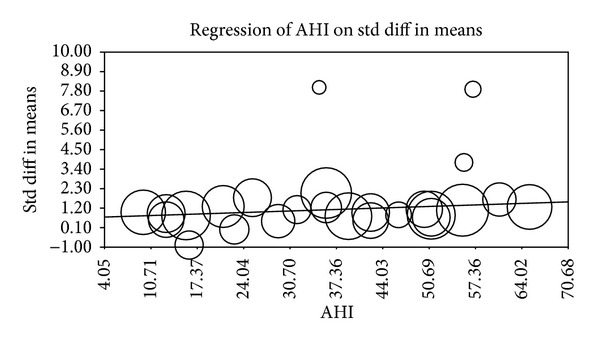
AHI metaregression.

**Figure 6:**
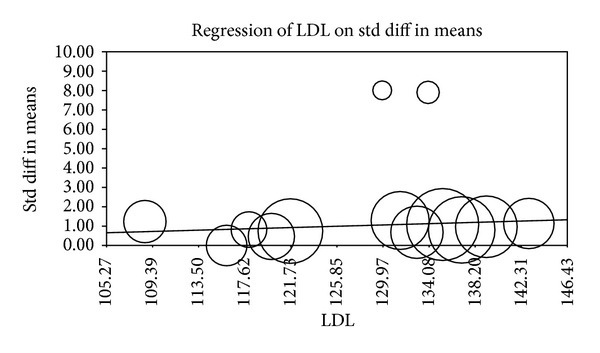
LDL metaregression.

**Figure 7:**
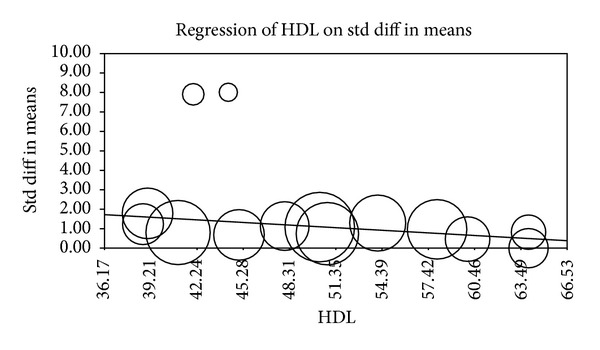
HDL metaregression.

**Figure 8:**
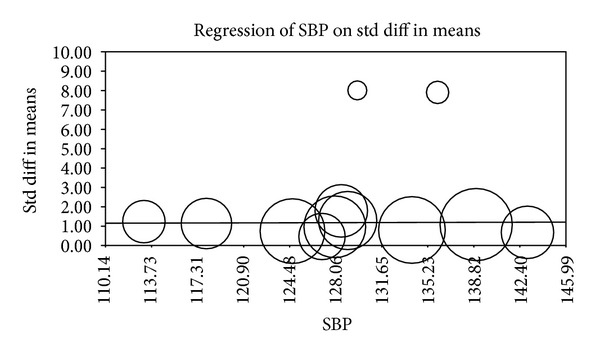
BP metaregression.

Multiple metaregression analyses were performed to evaluate the effect of age, gender, BMI, AHI, LDL cholesterol, HDL cholesterol, and systolic BP on CIMT when reported in manuscripts. Age (Beta 0.059, *P* = 0.0001), gender (Beta 0.010, *P* = 0.015), AHI (Beta 0.012, *P* = 0.0004), and HDL (Beta −0.04, *P* = 0.0004) were found to have modest but significant effect as a confounding factor (Table 2) (Figures 2, 3, 4, 5, 6, 7, and 8). 

## 4. Discussion

The existence of vascular inflammation in patients diagnosed with OSA has been supported by many studies. Such processes can cause atherosclerosis of vessels and contribute to the development of cardiovascular and cerebrovascular pathologies. Although atherosclerotic disease cannot be reliably detected by traditional risk assessment, CIMT can be used as a surrogate marker for the inflammatory process and allow for identification of subclinical atherosclerosis [6]. 

This meta-analysis shows that patients diagnosed with OSA display a statistically significant larger CIMT when compared to controls. There are conflicting results for the association between OSA and increased CIMT. The majority of studies reviewed for this analysis have found elevated CIMT in OSA patients when compared to controls and have also shown that overall thickness of the intima media increases with severity of sleep apnea. 

In their case-control studies, Lefebvre et al. [22], Meng et al. [29], Minoguchi et al. [30], Schulz et al. [34], Silvestrini et al. [35], Tanriverdi et al. [13], and Yun et al. [36] have demonstrated a direct relationship between increased CIMT and OSA by comparing 2 groups of subjects, 1 group with OSA and a control group. In each of these studies the OSA group of subjects had a significantly larger average CIMT when compared to that of the controls. 

A number of other studies have attained results that confirm the correlation between OSA and increased CIMT, while also demonstrating that CIMT values become even larger as the severity of OSA increases. Altin et al. [23] and Kaynak et al. [27] compared their controls to 2 groups of patients, one diagnosed with mild-moderate OSA and the other diagnosed with severe OSA. In both of these studies, it was determined that CIMT increased in the order of control, mild-moderate OSA, and severe OSA. Li and colleagues [28] divided their subjects into four groups and arrived at similar conclusions. The results they obtained determined that CIMT increased in the order of control, mild OSA, moderate OSA, and severe OSA. 

The metaregression analysis we performed to evaluate the effect of age, gender, BMI, AHI, LDL cholesterol, HDL cholesterol, and systolic BP on CIMT showed that age, gender, AHI, and HDL were significant as a confounding factor despite the modest effect (Figures 2–8) (Table 2). This is in agreement with the literature documenting the significance of these confounding factors in determining the CIMT. Güven et al. found that age and BMI were the most important independent determinants of carotid IMT in their study [24]. Howard et al. reported similar results that IMT measurements show a consistent progression with age and greater values among men [31]. Regarding severity of OSA Drager et al. 2005 [33] compared controls to patients diagnosed with mild-moderate OSA, and severe OSA. They reported that CIMT increased in the order of mild-moderate OSA, control, and severe OSA. Although the control group was determined to have a slightly larger CIMT when compared to the mild-moderate OSA group, the researchers determined that the difference in value was not statistically significant and concluded that IMT is correlated with OSA severity [33].

A study performed by Monneret et al. 2010 [26] obtained results with a similar discrepancy as previously mentioned. They compared the same groups as Drager et al. 2005 [33] and found that their controls and mild-moderate OSA patients had equal CIMT, which was less than that of severe OSA patients. These researchers determined that the CIMT values calculated for the controls and mild-moderate OSA group were not statistically significant and that CIMT was positively correlated with AHI [26]. 

Saletu and colleagues [32] compared CIMT values of their controls to three groups of patients diagnosed with different severities of OSA: mild, moderate, and severe. They determined that CIMT values increased in the order of control, mild OSA, severe OSA, and moderate OSA. The difference in CIMT values for the moderate and severe OSA groups does not appear to be statistically significant. Therefore, this study does not contradict the hypothesis that CIMT values are positively correlated with increasing severity of sleep apnea. 

Drager et al. 2010 [25] and Monneret et al. 2012 [37] performed separate studies to compare CIMT values in patients diagnosed with metabolic syndrome (MetS) with and without OSA. The results of both studies determined that CIMT was significantly increased in MetS patients diagnosed with OSA compared to MetS patients without OSA. The researchers in both studies reached the same conclusion that OSA is very common in MetS patients and that it plays an incremental role in the atherosclerotic burden and occurrence of vascular remodeling in this population [25, 37]. Data on blood glucose values and prevalence of Met syndrome were only available for insufficient number of studies; therefore, metaregression cannot be performed for this variable. Drager et al. 2009 [38] performed a study to analyze the combined effects of OSA and hypertension on early markers of atherosclerosis. They compared CIMT values for their controls to 2 groups of patients, one with OSA only and the other with OSA and hypertension. They found that CIMT values increased the order of control, OSA, and OSA + HTN. The researchers concluded that, when OSA is associated with HTN, additive effects are seen on markers of carotid atherosclerosis [38]. Data on prevalence of hypertension were only available for insufficient number of studies; therefore, metaregression cannot be performed for this variable.

There are multiple limitations of this meta-analysis that should be emphasized. It is very clear that the available literature relevant to this study is largely low-level evidence. A potential limitation is that we excluded all papers written in languages other than English, which could raise the possibility of publication bias. Also, we included only studies in which CIMT was evaluated by ultrasonography, even though there are other methods available to assess this value. 

Other limitations pertain to the methods of individual studies included in this analysis. There was a heterogeneity in the size of sample populations and also in the patient characteristics of the sample populations between studies. It is known that studies with positive results tend to get published while studies with negative results are less likely to be published in general, and since we only included data from published studies in our meta-analysis, we assume publication bias does exist.

Despite the limitations with this meta-analysis, it was reassuring that the majority of analyzed studies determined that OSA subjects display an increase in CIMT when compared to control individuals. Moreover, most of the studies that divided OSA patients into groups based on AHI found that CIMT is positively correlated with the severity of the disease. These results suggest that selection and sampling biases were not likely to be responsible for the observed associations. 

In summary, there appears to be evidence indicating increased CIMT in OSA patients when compared to control populations. Some of this effect is attributed to confounding factors as suggested by metaregression. These findings may explain the high occurrence of atherosclerosis contributing to cardiovascular and cerebrovascular pathologies in patients diagnosed with OSA. Future studies are needed to determine the reliability of CIMT as a disease-associated marker for atherosclerosis in OSA patients, to determine the correlation between CIMT and severity of OSA and to determine whether elevated CIMT due to atherosclerosis could be modified by therapeutic interventions for OSA. 

## Figures and Tables

**Table 1:** Included studies assessing CIMT thickness in adult OSA patients versus control.

Study	Study design	Size of study population	Outcome
Altin et al. 2005 [23]	Case-control	30 severe OSA 20 mild OSA 20 control	IMT values increase in the order of control, mild OSA, and severe OSA IMT values increase with severity of OSA
Güven et al. 2000 [24]	Case-control	15 severe OSA 15 mild-moderate OSA 12 control	IMT values increase in the order of mild-moderate OSA, control, and severe OSA
Drager et al. 2010 [25]	Case-control	25 OSA 27 OSA + HTN 22 control	IMT values increase in the order of control, OSA, OSA + HTN
Monneret et al. 2010 [26]	Case-control	51 OSA + Metabolic Syndrome 30 Metabolic Syndrome (control)	IMT values were increased in MetS + OSA compared to control
Kaynak et al. 2003 [27]	Case-control	36 severe OSA 41 mild-moderate OSA 37 control	IMT values increase in the order of control, mild-moderate OSA, and severe OSA IMT values increase with severity of OSA
Lefebvre et al. 2008 [22]	Case-control	40 OSA 20 control	IMT values were increased in OSA group compared to control
Li et al. 2009 [28]	Case-control	18 severe OSA 18 moderate OSA 16 mild OSA 18 control	IMT values increase in the order of control, mild OSA, moderate OSA, and severe OSA IMT values increase with severity of OSA
Meng et al. 2009 [29]	Case-control	75 OSA 48 control	IMT values were increased in OSA group compared to control
Minoguchi et al. 2005 [30]	Case-control	36 OSA 16 control	IMT values were increased in OSA group compared to control
Howard et al. 1993 [31]	Case-control	12 severe OSA 19 mild-moderate OSA 10 control	IMT values are equal between control and mild-moderate OSA, and increased in severe OSA
Saletu et al. 2008 [32]	Case-control	26 OSA + Metabolic Syndrome 9 Metabolic Syndrome (control)	IMT values were increased in MetS + OSA compared to control
Drager et al. 2005 [33]	Case-control	51 severe OSA 25 moderate OSA 27 mild OSA 44 control	IMT values increase in the order of control, mild OSA, severe OSA, and moderate OSA
Schulz et al. 2005 [34]	Case-control	35 OSA 35 control	IMT values were increased in OSA group compared to control
Silvestrini et al. 2002 [35]	Case-control	23 OSA 23 control	IMT values were increased in OSA group compared to control
Tanriverdi et al. 2006 [13]	Case-control	40 OSA 24 control	IMT values were increased in OSA group compared to control
Yun et al. 2010 [36]	Case-control	82 OSA 22 control	IMT values were increased in OSA group compared to control

**Table 2:** Metaregressions statistics for evaluated confounding factors.

Confounding factors	Slope	Intercept	*P* value
Age	0.059	−1.89	0.0001
Gender	0.010	0.331	0.015
BMI	0.016	0.653	0.39
AHI	0.012	0.642	0.0004
LDLc	0.016	−1.055	0.077
HDLc	−0.04	3.319	0.0004
Systolic BP	0.001	0.971	0.883

## References

[1] Young T, Palta M, Dempsey J, Skatrud J, Weber S, Badr S (1993). The occurrence of sleep-disordered breathing among middle-aged adults. *New England Journal of Medicine*.

[2] Young T, Skatrud J, Peppard PE (2004). Risk factors for obstructive sleep apnea in adults. *Journal of the American Medical Association*.

[3] Guilleminault C, Tilkian A, Dement WC (1976). The sleep apnea syndromes. *Annual Review of Medicine*.

[4] Somers VK, Dyken ME, Clary MP, Abboud FM (1995). Sympathetic neural mechanisms in obstructive sleep apnea. *Journal of Clinical Investigation*.

[5] Kato M, Roberts-Thomson P, Phillips BG (2000). Impairment of endothelium-dependent vasodilation of resistance vessels in patients with obstructive sleep apnea. *Circulation*.

[6] Cobble M, Bale B (2010). Carotid intima-media thickness: knowledge and application to everyday practice. *Postgraduate medicine*.

[7] Finn AV, Kolodgie FD, Virmani R (2010). Correlation between carotid intimal/medial thickness and atherosclerosis: a point of view from pathology. *Arteriosclerosis, Thrombosis, and Vascular Biology*.

[8] Kastelein JJP, de Groot E (2008). Ultrasound imaging techniques for the evaluation of cardiovascular therapies. *European Heart Journal*.

[9] Brenner DJ, Hall EJ (2007). Computed tomography-an increasing source of radiation exposure. *New England Journal of Medicine*.

[10] Sharma K, Blaha MJ, Blumenthal RS, Musunuru K (2009). Clinical and research applications of carotid intima-media thickness. *American Journal of Cardiology*.

[11] Chambless LE, Heiss G, Folsom AR (1997). Association of coronary heart disease incidence with carotid arterial wall thickness and major risk factors: the Atherosclerosis Risk in Communities (ARIC) study, 1987–1993. *American Journal of Epidemiology*.

[12] Geroulakos G, O’Gorman DJ, Kalodiki E, Sheridan DJ, Nicolaides AN (1994). The carotid intima-media thickness as a marker of the presence of severe symptomatic coronary artery disease. *European Heart Journal*.

[13] Tanriverdi H, Evrengul H, Kara CO (2006). Aortic stiffness, flow-mediated dilatation and carotid intima-media thickness in obstructive sleep apnea: non-invasive indicators of atherosclerosis. *Respiration*.

[14] Hozo SP, Djulbegovic B, Hozo I (2005). Estimating the mean and variance from the median, range, and the size of a sample. *BMC Medical Research Methodology*.

[15] Saletu M, Sauter C, Lalouschek W (2008). Is excessive daytime sleepiness a predictor of carotid atherosclerosis in sleep apnea?. *Atherosclerosis*.

[16] Drager LF, Bortolotto LA, Maki-Nunes C (2010). The incremental role of obstructive sleep apnoea on markers of atherosclerosis in patients with metabolic syndrome. *Atherosclerosis*.

[17] Drager LF, Bortolotto LA, Krieger EM, Lorenzi-Filho G (2009). Additive effects of obstructive sleep apnea and hypertension on early markers of carotid atherosclerosis. *Hypertension*.

[18] Monneret D, Tamisier R, Ducros V (2012). The impact of obstructive sleep apnea on homocysteine and carotid remodeling in metabolic syndrome. *Respiratory Physiology and Neurobiology*.

[19] Kashine S, Kishida K, Funahashi T (2010). Characteristics of sleep-disordered breathing in Japanese patients with type 2 diabetes mellitus. *Metabolism*.

[20] Wattanakit K, Boland L, Punjabi NM, Shahar E (2008). Relation of sleep-disordered breathing to carotid plaque and intima-media thickness. *Atherosclerosis*.

[21] Sackett DL, Straus SE, Richardson WS, Rosenberg W, Haynes RB (2000). *Evidence-Based Medicine-How to Practice and Teach EBM*.

[22] Lefebvre B, Pépin J-L, Baguet J-P (2008). Leukotriene B4: early mediator of atherosclerosis in obstructive sleep apnoea?. *European Respiratory Journal*.

[23] Altin R, Özdemir H, Mahmutyazicioğlu K (2005). Evaluation of carotid artery wall thickness with high-resolution sonography in obstructive sleep apnea syndrome. *Journal of Clinical Ultrasound*.

[24] Güven N, Tütüncü NB, Oto A, Erbås T (2000). Major determinants of the carotid intima-media thickness in type 2 diabetic patients: age and body mass index. *Endocrine Journal*.

[25] Drager LF, Bortolotto LA, Maki-Nunes C (2010). The incremental role of obstructive sleep apnoea on markers of atherosclerosis in patients with metabolic syndrome. *Atherosclerosis*.

[26] Monneret D, Pepin J, Godin-Ribuot D (2010). Association of urinary 15-F2t-isoprostane level with oxygen desaturation and carotid intima-media thickness in nonobese sleep apnea patients. *Free Radical Biology and Medicine*.

[27] Kaynak D, Göksan B, Kaynak H, Degirmenci N, Daglioglu S (2003). Is there a link between the severity of sleep-disordered breathing and atherosclerotic disease of the carotid arteries?. *European Journal of Neurology*.

[28] Li C, Zhang X, Liu H, Wang Z, Yin K (2009). Association among plasma interleukin-18 levels, carotid intima-media thickness and severity of obstructive sleep apnea. *Chinese Medical Journal*.

[29] Meng S, Fang L, Wang C, Wang L, Chen M, Huang X (2009). Impact of obstructive sleep apnoea on clinical characteristics and outcomes in patients with acute coronary syndrome following percutaneous coronary intervention. *The Journal of international medical research*.

[30] Minoguchi K, Yokoe T, Tazaki T (2005). Increased carotid intima-media thickness and serum inflammatory markers in obstructive sleep apnea. *American Journal of Respiratory and Critical Care Medicine*.

[31] Howard G, Sharrett AR, Heiss G (1993). Carotid artery intimal-medial thickness distribution in general populations as evaluated by B-mode ultrasound. *Stroke*.

[32] Saletu M, Sauter C, Lalouschek W (2008). Is excessive daytime sleepiness a predictor of carotid atherosclerosis in sleep apnea?. *Atherosclerosis*.

[33] Drager LF, Bortolotto LA, Lorenzi MC, Figueiredo AC, Krieger EM, Lorenzi-Filho G (2005). Early signs of atherosclerosis in obstructive sleep apnea. *American Journal of Respiratory and Critical Care Medicine*.

[34] Schulz R, Seeger W, Fegbeutel C (2005). Changes in extracranial arteries in obstructive sleep apnoea. *European Respiratory Journal*.

[35] Silvestrini M, Rizzato B, Placidi F, Baruffaldi R, Bianconi A, Diomedi M (2002). Carotid artery wall thickness in patients with obstructive sleep apnea syndrome. *Stroke*.

[36] Yun CH, Jung KH, Chu K (2010). Increased circulating endothelial mocroparticles and carotid atherosclerosis in obstructive sleep apnea. *Journal of Clinical Neurology*.

[37] Monneret D, Tamisier R, Ducros V (2012). The impact of obstructive sleep apnea on homocysteine and carotid remodeling in metabolic syndrome. *Respiratory Physiology and Neurobiology*.

[38] Drager LF, Bortolotto LA, Krieger EM, Lorenzi-Filho G (2009). Additive effects of obstructive sleep apnea and hypertension on early markers of carotid atherosclerosis. *Hypertension*.

